# Non-linear associations of maternal pre-pregnancy body mass index with risk of stillbirth, infant, and neonatal mortality in over 28 million births in the USA: a retrospective cohort study

**DOI:** 10.1016/j.eclinm.2023.102351

**Published:** 2023-12-02

**Authors:** Hannah V. Thornton, Rosie P. Cornish, Deborah A. Lawlor

**Affiliations:** a Population Health Sciences, Bristol Medical School, University of Bristol, Bristol, UK; b MRC Integrative Epidemiology Unit at the University of Bristol, Bristol, UK; c Bristol National Institute for Health Research Biomedical Research Centre, University of Bristol, Bristol, UK

**Keywords:** Body mass index, Stillbirth, Infant mortality, Neonatal mortality

## Abstract

**Background:**

Higher maternal pre-pregnancy body mass index (BMI) has been associated with higher risk of stillbirth, infant and neonatal mortality. Studies exploring underweight have varied in their conclusions. Our aim was to examine the risk of stillbirth, infant and neonatal mortality across the BMI distribution and establish a likely healthy BMI range.

**Methods:**

In this retrospective cohort study, we used publicly available datasets (covering 1st January 2014 to 31st December 2021) from the US National Center for Health Statistics National Vital Statistics System. All births were eligible; analyses included those with non-missing data. Fractional polynomial multivariable logistic regression was used to examine the associations of maternal pre-pregnant BMI with stillbirth (birth with no signs of life at ≥24 weeks), infant mortality (death of a live born baby aged <365 days) and neonatal mortality (death of a live born baby aged <28 days).

**Findings:**

There were 77,896/28,310,154 (2.8 per 1000 births) stillbirths, 143,620/28,231,807 (5.1 per 1000 live births) infant deaths and 94,246/28,231,807 (3.3 per 1000 live births) neonatal deaths among complete cases. Mean (SD) BMI was 27.1 kg/m^2^ (6.7 kg/m^2^). We found non-linear associations between BMI and all three outcomes; risk was elevated at both low and high BMIs although, for stillbirth, the increased risk at low BMI was less marked than for infant/neonatal mortality. The lowest risk was at a BMI of 21 kg/m^2^ for infant and neonatal mortality and 19 kg/m^2^ for stillbirth.

**Interpretation:**

Public health messaging for preconception and postnatal care should focus on healthy weight to maximise maternal and child health, and not focus solely on maternal overweight or obesity.

**Funding:**

10.13039/501100000781European Research Council, US National Institute of Health, 10.13039/501100000265UK Medical Research Council and 10.13039/501100000272National Institute for Health and Care Research.

**Research in context**

**Evidence before this study**

We carried out a Medline search (1st January 1946 to 14th December 2022) with no language restrictions, using used a combination of the following: body mass index; BMI; obesity; overweight; underweight; weight; low weight; low BMI; maternal; stillbirth; infant death; infant mortality; neonatal death; neonatal mortality; birth outcome. We identified five systematic reviews and four additional primary studies published after the most recent review; these consistently showed an association of increased risk of stillbirth, neonatal mortality and infant mortality in women with overweight or obesity whereas the results for underweight were inconsistent. Two of the reviews found evidence for non-linear associations, but again the results were inconsistent for low BMI. On the whole, the quality of studies included in the reviews was assessed as high.

**Added value of this study**

In this study of over 28 million births, we examine non-linear associations of maternal pre-pregnant BMI with stillbirth, infant and neonatal mortality using BMI as a continuous variable across the whole BMI distribution. This is the largest study to date (the largest systematic reviews pooled data on 10.8 million births for infant mortality, 3.8 million for neonatal mortality and 3.3 million for stillbirth), providing sufficient sample size to investigate the risk of these relatively rare outcomes across the whole maternal BMI range, in which we adjust for pre-specified confounders and include all women from a whole (US) population. We also compare results across three different definitions of stillbirth currently used in different regions of the world.

**Implications of all the available evidence**

The relationship between maternal pre-pregnancy BMI and risk of stillbirth infant and neonatal mortality is non-linear, with increased risk at both low and high BMIs. Public health messaging for preconception and postnatal care should focus on both maternal over and underweight.

## Introduction

Stillbirth (birth with no signs of life from between 20 and 28 weeks of gestation, with definitions varying between populations and institutions and hence studies), infant mortality (death of a live born baby aged <365 days) and neonatal mortality (death of a live born baby aged <28 days, a subgroup of infant death) are devastating events with severe repercussions for families and healthcare services. Globally, an estimated 2.6 million third trimester (i.e., ≥28 weeks) stillbirths occurred in 2015 and, in 2021 over 3.7 million children died before the age of 1.^1^^,^^2^

Internationally, obesity has almost tripled since 1975 and the most recent WHO data shows that an estimated 25% of adults had obesity in 2020.^3^ The prevalence of underweight is slowly falling, yet an estimated 9.4% of all females had underweight in 2016.^4^ Whilst higher maternal body mass index (BMI) is consistently associated with a higher risk of stillbirth, neonatal mortality and infant mortality, there is less certainty over the associations of underweight with these outcomes^1^^,^^5^ There is an established higher risk of foetal growth restriction and low birth weight in underweight women, and a strong relationship between foetal growth restriction and stillbirth and infant mortality.^6^ One might therefore expect an increased risk of stillbirth, neonatal and infant mortality in women with underweight.

Of the five previous systematic reviews^5^^,^^7^, ^8^, ^9^, ^10^ and four subsequent primary studies^11^, ^12^, ^13^, ^14^ on the relationship of pre-pregnancy maternal BMI on stillbirth, infant or neonatal mortality, all reported results using BMI categories, with three (including two of the reviews) not examining the association of underweight with these outcomes. These nine studies all reported analyses supporting an increased risk of all three outcomes among women with overweight or obesity, with less consistency regarding underweight. Two of the reviews used information from the BMI categories to estimate a relative risk for a unit increase in BMI and to examine non-linear associations, one with infant and neonatal mortality,^10^ one with all three outcomes.^5^ Both found evidence for non-linearities but, again, these were not consistent, with one indicating a decreasing risk of infant mortality with lower BMIs^10^ but the other finding a very slight increase below a BMI of around 20 kg/m^2^ but a decreasing risk of stillbirth with lower BMIs. The largest meta-analyses of stillbirth, infant mortality and neonatal mortality included 3.3 million, 10.8 million and 3.8 million births, respectively.

Understanding the relationships between maternal underweight, as well as overweight and obesity, and stillbirth, neonatal and infant mortality might enable us to identify a “safe” target weight for women considering pregnancy; this has not yet been identified.^5^ This could help parents and healthcare providers make informed decisions about antenatal monitoring, delivery and infant care for mothers whose weight (whether under- or overweight) might confer increased risk of stillbirth and infant mortality.

The aim of this study was to explore whether there were non-linear associations of maternal pre-pregnant BMI with stillbirth, neonatal and infant mortality in order to identify a “healthy” BMI for these outcomes. We add to existing literature by analysing the largest population to date, over 28 million recorded births in the United States (US) between 2014 and 2021, providing sufficient sample size to investigate the risk of these relatively rare outcomes across the whole maternal BMI range.

## Methods

### Dataset

This study used publicly available US National Center for Health Statistics (NHCS) National Vital Statistics System datasets (covering the period 1st January 2014 to 31st December 2021, inclusive), which contain details of all births registered in the USA.^15^ For stillbirths we used the Birth and Fetal Death Data Files; for infant, including neonatal mortality, we used the Period/Cohort Linked Birth-Infant Death Datafiles. The Fetal Death files exclude all terminations.^15^ This timeframe was selected because maternal pre-pregnancy BMI was included in the foetal deaths data from 2014 onwards and, at the time of analysis, foetal and infant death data were available up to and including 2021. Each record in these datasets relates to a live birth or foetal death, rather than a pregnancy and there are no pregnancy or person-level identifiers in the dataset. To identify pregnancies, we matched multiple births occurring close in time in which birth and maternal characteristics were the same.

### Ethics

These analyses used publicly available data containing no identifying information; no ethical approval was required for this study. We have adhered to the Vital Statistics Data User Agreement in the use of these data. The NHCS are bound by federal regulations to ensure data confidentiality. NCHS provides respondents with a description of the intended purpose(s) of the data they provide and informed consent is assumed if the respondent gives the requested information.

### Exposure

Maternal pre-pregnancy height and weight were self-reported by the women at the time of birth via the following questions: What is your height? What was your pre-pregnancy weight? That is, your weight immediately before you became pregnant with this child? BMI was calculated from these. BMI was used in its continuous form in the primary analysis. For the descriptive and secondary analysis, we used eight World Health Organisation (WHO) BMI categories, including categories of underweight (severe underweight: <16 kg/m^2^, moderate underweight: 16–16.9 kg/m^2^, mild underweight: 17–18.49 kg/m^2^); normal: 18.5–24.9 kg/m^2^; overweight 25–29.9 kg/m^2^; obesity (class-I: 35–34.9 kg/m^2^, class-II: 35–39.9 kg/m^2^ and class-III: ≥40 kg/m^2^).

### Outcomes

Gestational age at which a foetal death is classified as a stillbirth varies internationally. The WHO define stillbirth as a baby born with no signs of life at ≥28 weeks’ gestation. In many European countries a threshold of gestational age of ≥22 weeks is used; 24 weeks is used in the United Kingdom and 20 weeks in the US.^16^ To enable comparison, we report results using three different definitions. For the main results we used ≥24 weeks’ gestation, with results using ≥20 and ≥28 weeks also reported. WHO definitions were used for infant mortality (death of a live born baby aged <365 days) and neonatal mortality (death of a live born baby aged <28 days).

### Potential confounders

We defined cofounders as any factors that are known or plausible causes of both the exposure (BMI) and outcomes (stillbirth and infant, including neonatal, mortality), as per best practice.^17^ The following variables were considered to be confounders, based on this definition: maternal age at birth (categorised as: <20, 20–24, 25–29, 30–34, 35–39, 40+ years); maternal education (<high school, high school, college, no degree, degree/higher); maternal ethnic group (White, Black, American Indian/Alaskan Native, Asian, Native Hawaiian/Pacific Islander, Mixed race); smoking status (non-smoker, stopped in early pregnancy, smoked throughout pregnancy); any other live birth in the previous 12 months (yes, no); parity (0, 1, 2, 3, ≥4); multiple birth (yes/no).

### Potential mediators

Our main aim was to determine the non-linear association of BMI with mortality outcomes and not to dilute this by adjusting away potential mediators though which BMI relates to mortality. We have undertaken exploratory analyses of the following potential mediators on the causal pathway between pre-pregnancy BMI and our outcomes of interest: diagnoses of gestational diabetes, gestational hypertension, and pre-eclampsia, delivery of a small or large for gestational age (SGA and LGA) infant, defined as below the 10th percentile and above the 90th percentile, respectively, and, for infant and neonatal mortality, preterm birth (<37 completed weeks gestation).

### Statistical analysis

We compared characteristics of the analysis sample (complete cases) with the whole sample to identify any patterns of missingness. We hypothesised that BMI and some of the confounders, particularly ethnicity and smoking, might plausibly be missing not at random (specifically, more likely to be missing if individuals had either a high or low BMI, were from an ethnic minority group, or were a smoker). Thus, we decided a priori to use a complete case analysis because in this situation (covariates missing not at random), multiple imputation would produce bias whereas a complete case logistic regression produces unbiased estimates of the exposure-outcome association unless the chance of being a complete case depends jointly on the exposure and outcome,^18^^,^^19^ which we thought unlikely. For the primary analysis, we used multivariable logistic regression using fractional polynomials^20^ with up to three powers of BMI (in its continuous form) to explore potential non-linear relationships between pre-pregnant BMI and each of the three outcomes (Further details regarding fractional polynomial models are given in the Supplementary Materials). Robust standard errors were used to take account of non-independence of outcomes of multiple births. We describe the overall shape of associations from these fractional polynomial regression analyses and, where there was evidence of a pre-pregnant BMI with lowest risk, we estimated that BMI value using differentiation and used bootstrapping to obtain its confidence interval. In a secondary analysis, we used BMI categories in a logistic regression. We then fitted further models to explore potential mediation of both BMI as a continuum in the fractional polynomials and BMI as categories. For each of these, the first mediation model included all confounders and gestational diabetes, gestational hypertension and pre-eclampsia; the second mediation model additionally included SGA and LGA, and the third (for infant and neonatal mortality only) additionally included preterm birth. All statistical analyses were carried out in Stata. Stata’s marginscontplot command was used to produce the plots of predicted risk; this gives adjusted predicted risks for each BMI value averaged over the confounders. Stata code used for the analysis is provided in the Supplementary Materials. We adhered to STROBE reporting guidelines when writing this manuscript.

### Role of the funding source

The funders of the study had no role in study design, data collection, data analysis, data interpretation, or writing of the report. RPC and HVT had access to the dataset. All authors had final responsibility for the decision to submit for publication.

## Results

**Fig. 1:**
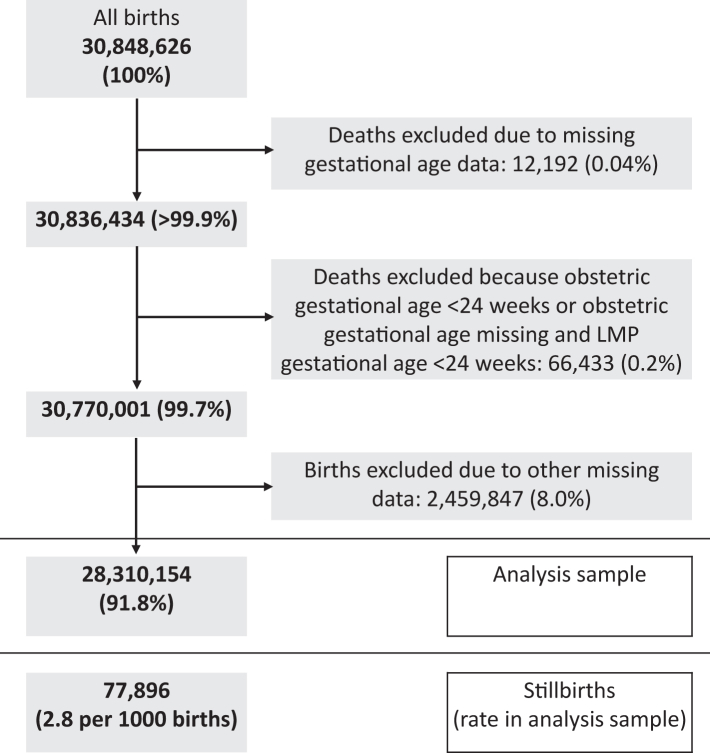
Stillbirth data inclusion flowchart (stillbirths after 24 weeks gestation).

**Fig. 2:**
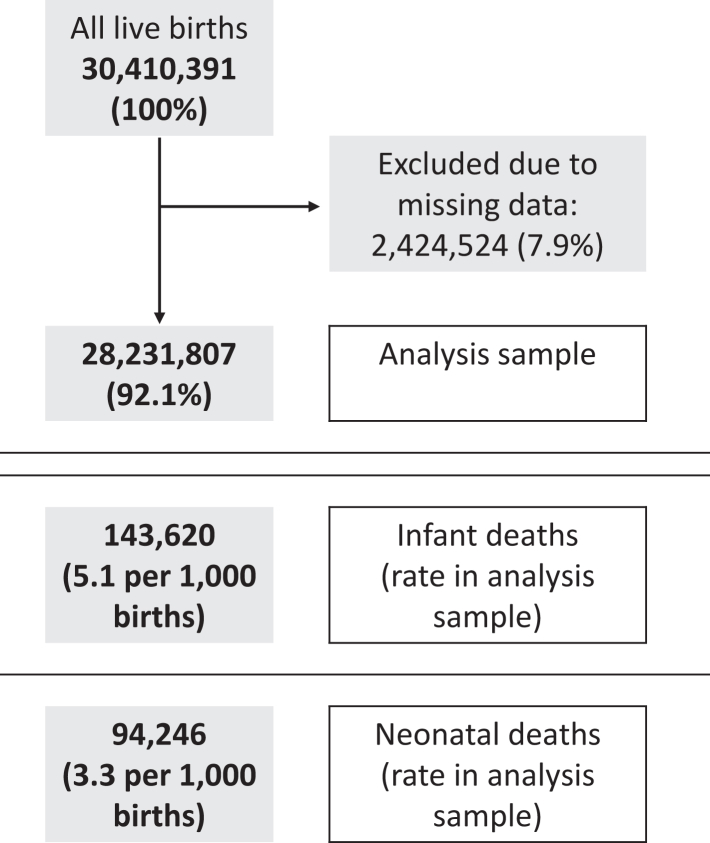
Infant mortality data inclusion flowchart.

The stillbirth dataset (24-week definition) contained 30,848,626 birth records of which 28,310,154 (92%) had no missing data (Fig. 1); the infant mortality dataset contained 30,410,391 birth records of which 28,231,807 (92%) had no missing data (Fig. 2). Among the complete case (i.e., no missing data) analysis samples, there were 77,896 (2.8 per 1000 births) stillbirths at or after 24 completed weeks, 143,620 (5.1 per 1000 live births) infant deaths, and 94,246 (3.3 per 1000 live births) neonatal deaths. Stillbirth rates were 4.3 and 2.1 per 1000 births with gestational age thresholds of 20 and 28 weeks, respectively. Stillbirth, infant and neonatal mortality rates were less common in the complete case sample compared to the whole sample (2.8 vs 3.7 per 1000 births for stillbirth, 3.3 vs 3.8 per 1000 live births for neonatal mortality and 5.1 vs 5.6 per 1000 live births for infant mortality), with the distribution of all other variables being similar (Tables 1 and 2).

**Table 1:** Characteristics of whole sample compared to analysis sample: stillbirth dataset (24-week definition).

Characteristic	Whole sample N = 30,836,434^a^	Analysis sample N = 28,310,154
**Stillbirth**	113,382 (3.7 per 1000 births)	77,896 (2.8 per 1000 births)
**Maternal pre-pregnant BMI**		
<18.5	969,965 (3%)	921,400 (3%)
18.5–24.9	12,700,637 (43%)	12,087,906 (43%)
25–29.9	7,890,244 (26%)	7,481,922 (26%)
30–34.9	4,463,384 (15%)	4,230,384 (15%)
35–39.9	2,202,143 (7%)	2,091,080 (7%)
40+	1,575,503 (5%)	1,497,462 (5%)
**Maternal age**		
<20	1,570,344 (5%)	1,473,528 (5%)
20–24	6,092,652 (20%)	5,674,172 (20%)
25–29	8,865,309 (29%)	8,187,278 (29%)
30–34	8,812,236 (29%)	8,056,181 (28%)
35–39	4,475,028 (15%)	4,023,408 (14%)
40+	1,020,865 (3%)	895,587 (3%)
**Maternal education**		
<High school	3,963,621 (13%)	3,625,442 (13%)
High school	7,764,242 (26%)	7,246,455 (26%)
College, no degree	8,606,952 (29%)	8,111,285 (29%)
Degree/higher	9,854,298 (33%)	9,326,972 (33%)
**Maternal ethnicity**		
White	22,626,111 (74%)	21,030,040 (74%)
Black	4,866,591 (16%)	4,378,027 (15%)
American Indian/Alaskan Native	297,076 (1%)	271,594 (1%)
Asian	2,014,500 (7%)	1,814,718 (6%)
Native Hawaiian/Pacific Islander	101,729 (0.3%)	84,310 (0.3%)
Mixed race	789,973 (3%)	731,465 (3%)
**Smoking status**		
Non-smoker	27,738,472 (91%)	25,834,705 (91%)
Stopped in early pregnancy	927,226 (3%)	879,982 (3%)
Smoked throughout pregnancy	1,724,205 (6%)	1,595,467 (6%)
**Parity**		
0	11,957,533 (39%)	11,375,293 (40%)
1	9,769,550 (32%)	8,849,605 (31%)
2	5,183,379 (17%)	4,666,799 (16%)
3	2,243,876 (7%)	2,006,080 (7%)
4+	1,610,164 (5%)	1,412,377 (5%)
**Other live birth in previous 12 months** (=yes)	259,362 (1%)	243,905 (1%)
**Multiple birth** (=yes)	1,044,739 (3%)	920,112 (3%)

a Denominators vary because not all variables are complete.

**Table 2:** Characteristics of whole sample compared to analysis sample: infant and neonatal mortality dataset—figures are n (%) except where otherwise indicated.

Characteristic	Whole sample N = 30,656,331^a^	Analysis sample N = 28,231,807
**Infant death**	170,519 (5.6 per 1000 live births)	143,620 (5.1 per 1000 live births)
**Neonatal death**	115,081 (3.8 per 1000 live births)	94,246 (3.3 per 1000 live births)
**Maternal pre-pregnant BMI (kg/m**^**2**^**)**		
<18.5	965,589 (3%)	919,222 (3%)
18.5–24.9	12,648,764 (43%)	12,061,051 (43%)
25–29.9	7,850,215 (26%)	7,461,304 (26%)
30–34.9	4,435,868 (15%)	4,216,286 (15%)
35–39.9	2,186,395 (7%)	2,083,257 (7%)
40+	1,562,224 (5%)	1,490,687 (5%)
**Maternal age**		
<20	1,559,003 (5%)	1,468,534 (5%)
20–24	6,056,802 (20%)	5,657,662 (20%)
25–29	8,817,394 (29%)	8,165,938 (29%)
30–34	8,765,856 (29%)	8,036,371 (28%)
35–39	4,446,066 (15%)	4,011,716 (14%)
40+	1,011,210 (3%)	891,586 (3%)
**Maternal education**		
<High school	3,939,160 (13%)	3,612,786 (13%)
High school	7,712,845 (26%)	7,220,510 (26%)
College, no degree	8,565,064 (29%)	8,089,785 (29%)
Degree/higher	9,819,116 (33%)	9,308,726 (33%)
**Maternal ethnicity**		
White	22,515,888 (74%)	20,978,128 (74%)
Black	4,817,283 (16%)	4,358,096 (15%)
American Indian/Alaskan Native	295,045 (1%)	270,564 (1%)
Asian	2,006,194 (7%)	1,811,268 (6%)
Native Hawaiian/Pacific Islander	100,633 (0.3%)	83,878 (0.3%)
Mixed race	786,843 (3%)	729,873 (3%)
**Smoking status**		
Non-smoker	27,598,069 (91%)	25,767,341 (91%)
Stopped in early pregnancy	922,007 (3%)	877,179 (3%)
Smoked throughout pregnancy	1,709,645 (6%)	1,587,287 (6%)
**Parity**		
0	11,889,744 (39%)	11,343,249 (40%)
1	9,727,899 (32%)	8,831,410 (31%)
2	5,153,744 (17%)	4,653,401 (16%)
3	2,227,281 (7%)	1,998,666 (7%)
4+	1,593,087 (5%)	1,405,081 (5%)
**Other live birth in previous 12 months** (=yes)	255,283 (1%)	242,340 (1%)
**Multiple birth** (=yes)	1,030,791 (3%)	915,583 (3%)

a Denominators vary because not all variables are complete.

**Fig. 3:**
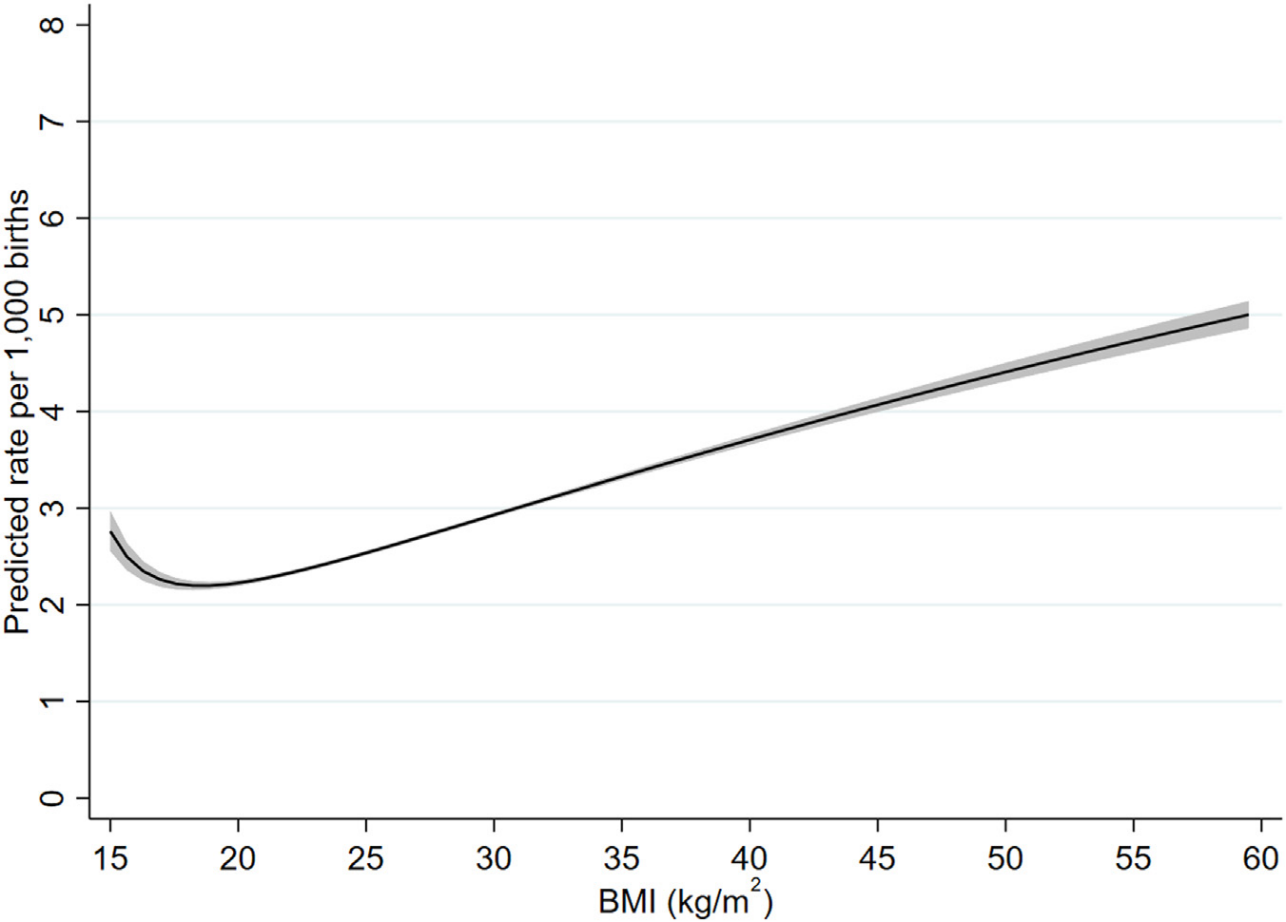
Adjusted rate of stillbirth by maternal pre-pregnant BMI (kg/m^2^) (shaded area indicates 95% confidence interval).

**Fig. 4:**
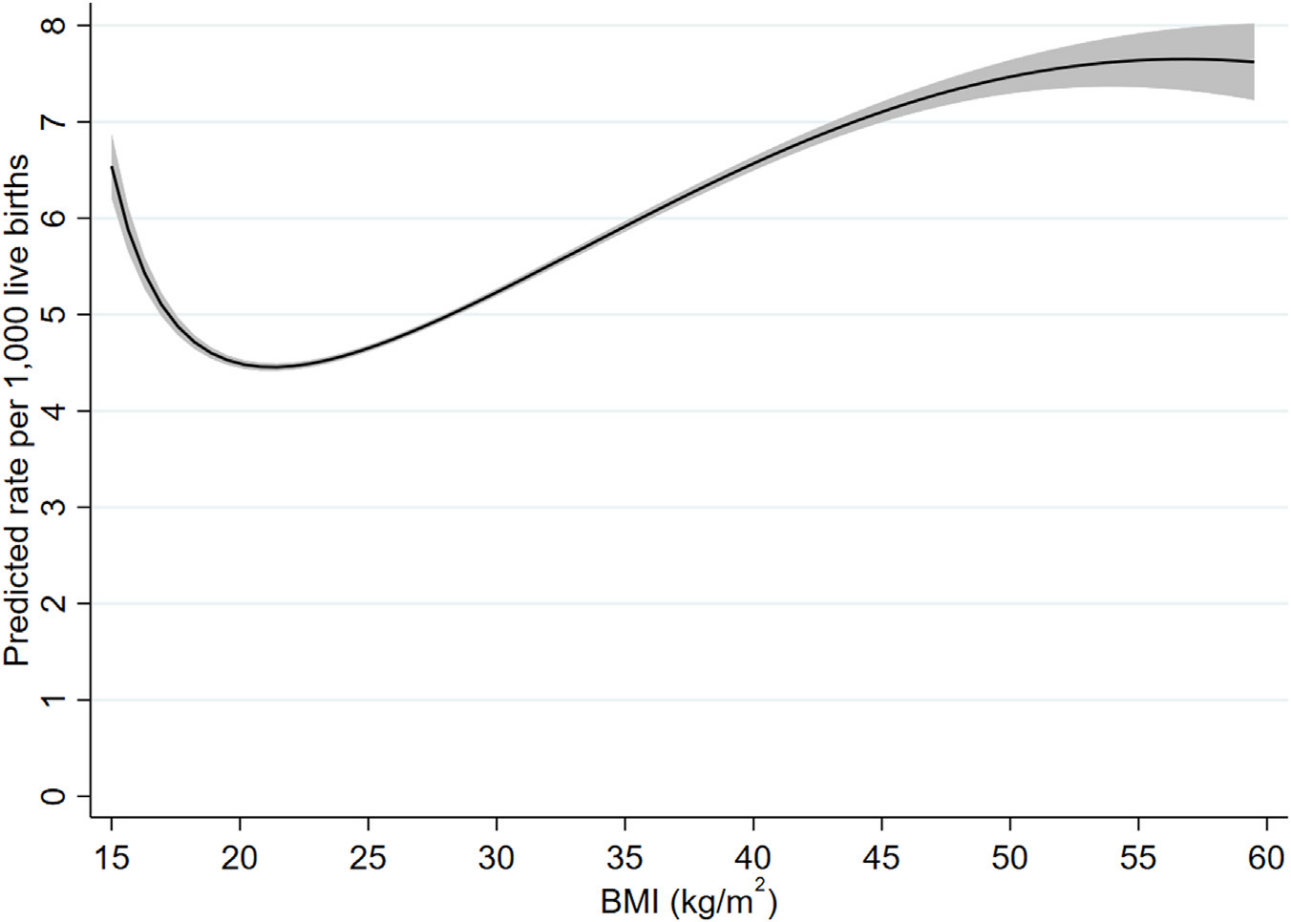
Adjusted rate of infant mortality by maternal pre-pregnant BMI (kg/m^2^) (shaded area indicates 95% confidence interval).

**Fig. 5:**
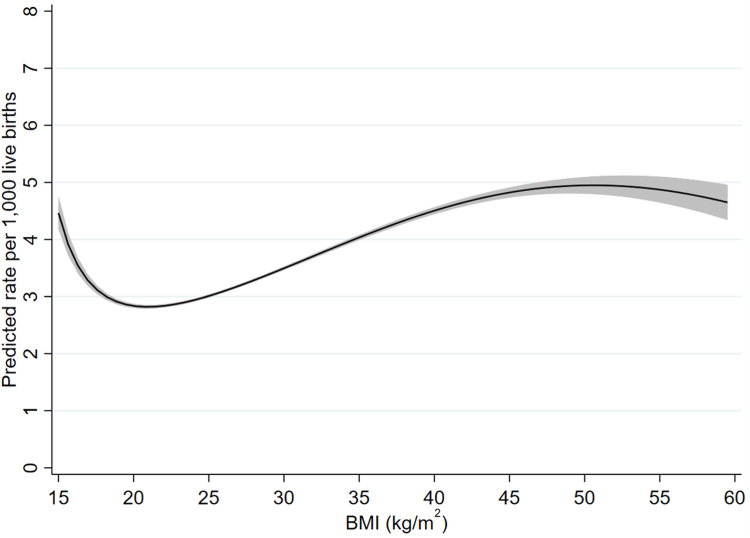
Adjusted rate of neonatal mortality by maternal pre-pregnant BMI (kg/m^2^) (shaded area indicates 95% confidence interval).

Details of the fractional polynomial models, including selection of the final models, are given in the Supplementary Text. For all three outcomes the best fitting two-degree model was a better fit than the best fitting one-degree model; for infant and neonatal mortality the three-degree model was a better fit than the two-degree one, whereas for stillbirth there was no clear difference between the two- and three-degree models. We therefore used three-degree models for infant and neonatal mortality and a two-degree model for stillbirth (Supplementary Text and Supplementary Figures S1 and S2). Fig. 3, Fig. 4, Fig. 5 give predicted risks (adjusted for confounders) across the BMI range obtained from these models. For all three outcomes there was evidence for a j-shaped curve, with an increased risk at very low maternal pre-pregnant BMIs as well as higher BMIs. For infant and neonatal mortality, the curve started to flatten at BMIs above about 50 kg/m^2^ and the estimated risk slightly decreased at the very highest BMIs, although the confidence intervals were quite wide at this extreme. The increased risk among underweight women was more marked for infant and neonatal mortality than for stillbirths, with the magnitude of the risk for a BMI of around 16 kg/m^2^ being similar to that at a BMI of between 30 and 35 kg/m^2^ for infant and neonatal mortality but about 22–23 kg/m^2^ for stillbirth (Fig. 3, Fig. 4, Fig. 5). The maternal BMI at which the predicted risk was lowest was 18.6 kg/m^2^ (95% CI: 16.0, 22.1 kg/m^2^) for stillbirth, 21.3 kg/m^2^ (21.2, 21.4 kg/m^2^) for infant mortality, and 21.2 kg/m^2^ (21.1, 21.3 kg/m^2^) for neonatal mortality.

The overall pattern of association for stillbirth remained similar when thresholds of 20 and 28 completed weeks of gestation were used, although predicted risks of stillbirth, across the distribution of BMI levels, were higher when a 20-week threshold was used (compared to 24 weeks) and lower when a 28-week threshold was used. In addition, the increased risk of stillbirth for low BMIs was more marked with a 20-week threshold than with the 24-week or 28-week threshold and the increase with higher BMIs was also more marked (Supplementary Figure S3).

Unadjusted risks of stillbirth, infant mortality and neonatal mortality using BMI categories are given in Supplementary Tables S1 and S2 and adjusted odds ratios given in Supplementary Table S3. Adjusted odds ratios for stillbirths using the 20-week and 28-week threshold are given in Supplementary Table S4. The patterns broadly matched those shown in Fig. 3, Fig. 4, Fig. 5, with an increased risk of infant and neonatal mortality across all underweight categories whereas, for stillbirth, the risk compared to women with normal weight was only elevated among those with severe and, to a lesser extent, moderate underweight. For all three outcomes risk was higher in women with overweight and with all categories of obesity. Similarly, the patterns seen for the different definitions of stillbirth match those seen in Supplementary Figure S3.

Including gestational diabetes, gestational hypertension and pre-eclampsia in the models as potential mediators made little difference to the patterns for all outcomes; neither did additionally adjusting for SGA and LGA for infant and neonatal mortality. The increased risk among underweight women was no longer present for stillbirth after adjusting for SGA and LGA and no longer present for infant and neonatal mortality after adjusting for preterm birth (Supplementary Figures S4 and S5; Supplementary Table S5).

## Discussion

In this study of over 28 million births, we have provided evidence for j-shaped associations between maternal pre-pregnant body mass index and stillbirth, infant mortality and neonatal mortality, with an increased risk among both underweight and overweight/obese women. This increased risk for underweight women was more marked for infant and neonatal mortality and only very slight for stillbirth. The lowest predicted risk occurred at a BMI of just over 21 kg/m^2^ for infant and neonatal mortality and at a BMI of just under 19 kg/m^2^ for stillbirth. Whilst preconceptual and antenatal advice now focuses on avoiding overweight and obesity,^21^ our results demonstrate that in contemporary high-income populations who have experienced several decades of the obesity epidemic, stillbirth, neonatal and infant mortality risk are higher in women with both higher and lower BMI. Taken together, the confidence intervals across all three outcomes from our study suggest that women with pre-pregnancy BMI below 16 kg/m^2^ or above 22.1 kg/m^2^ are at increased risk of all three outcomes, with values around 21 kg/m^2^ likely associated with lowest risk of all three.

Our aim was to explore associations of total BMI with stillbirth, infant and neonatal mortality (i.e., not adjusted for potential mediating factors). In additional exploratory analyses we did not see evidence that gestational diabetes, gestational hypertension or pre-eclampsia mediated this association. There was evidence that the increased risk of stillbirth at lower levels of BMI was potentially mediated by SGA and LGA and that increased infant and neonatal mortality at lower BMI were mediated by preterm birth. However, these results should be treated with caution as any confounding between the mediator and outcome could result in bias in either direction.^22^ That said, our findings reflect evidence that, for example, SGA is a key determinant of stillbirth, to the extent that it is used as a proxy in randomised controlled trials for stillbirth because of the difficulties of achieving the sample size in a trial that would be necessary for precise effect estimates with this rare outcome.^23^

Previous studies have consistently found an increased risk of these outcomes with overweight and obesity categories but the results for underweight have been less consistent. In the most recent and largest meta-analysis, underweight was found to be associated with a decreased risk of infant mortality.^10^ However, this result was dominated by one large study (receiving 83% of the weight) in which the odds ratios were adjusted for gestational age and small for gestational age. These factors are likely to be on the causal pathway between maternal BMI and infant mortality, thus meaning that this is not testing the overall effect of underweight, much of which is likely due to the impact of maternal BMI on preterm and small for gestational age.^24^ To our knowledge, only three previous studies have analysed the association between pre-pregnant BMI and these outcomes using maternal BMI as a continuous variable. One examined the association with foetal or infant death as a combined outcome and found evidence for a V-shaped relationship with a minimum at a BMI of 23 kg/m^2^.^25^ Another used restricted cubic splines to examine the association with a number of adverse pregnancy outcomes, including stillbirth; they found that the risk increased across the range of BMI.^26^ In both these studies, the numbers of women classified as underweight were relatively small and therefore confidence intervals were wide. The third study investigated whether there was evidence for a quadratic relationship between maternal BMI and stillbirth in different subgroups of ethnicity, parity and gestational age but women with underweight were excluded due to small numbers.^27^ We have added to previous work by determining the pattern of association across the whole maternal distribution in a large sample which includes over 800,000 women with underweight and deriving BMI levels at which risk for all three of stillbirth, infant death and its subgroup of neonatal death are lowest.

Key strengths of this study include the large sample size and the fact that we have explored non-linear associations with BMI as a continuous variable. In secondary analyses we explored the associations of BMI categories with each of the three outcomes as we considered this useful for clinical/public health practitioners and for comparison with other studies. However, whilst we had sufficient power to explore associations with all eight BMI categories, it should be noted that in analyses with BMI as a categorical variable, results reflect the “average” risk within a BMI category (e.g., the odds ratio for the overweight category compares the average odds among women with BMI values between 25 and 29.9 kg/m^2^ to the average odds among those with a BMI between 18.5 and 24.9 kg/m^2^), which masks variation in risk within each category and makes it impossible to define the BMI at which risk is minimised. This was why we used BMI in its continuous form in our primary analyses.

There was complete data on over 91% of participants and the distribution of most characteristics was similar among the whole sample and complete cases. Data were less complete for stillbirths and births that resulted in infant deaths. As a result, the rates of the three outcomes were lower in complete cases compared to those not restricted to individuals with complete data. A complete case logistic regression, as used here, will give unbiased estimates of the exposure odds ratio unless missingness depends jointly on the exposure and outcome^18^^,^^19^ (i.e., in this case unless the relationship between BMI and the likelihood of it being missing were different among individuals whose infant subsequently died compared to those whose infant survived/among live births and stillbirths). We thought this was unlikely in the case of infant (including neonatal) mortality but possible for stillbirth because BMI and several of the confounders were reported by the mother at the time of (still)birth. However, with over 91% complete data, any resulting bias is likely to be small^28^ and, because (as stated previously) we believed that BMI and some confounders might be missing not at random, meaning that multiple imputation could have led to bias, we felt that a complete case analysis was the most appropriate approach. Thus, although the absolute risks of the three outcomes would be underestimated in the complete case data, we think missing data will have had a negligible impact on the overall pattern of association with BMI. Weight and height, as well as some confounders, were self-reported and thus subject to misreporting and recall (measurement) error. Although agreement between reported and measured weight tend to be relatively high,^29^ women with overweight and obesity are more likely to underreport their weight and women with underweight to overreport their weight, resulting in greater measurement error at very low and very high BMIs.^29^^,^^30^ This would tend to result in the risk of our outcomes being overestimated at the extremes of BMI. However, in this population, this error at the extremes had little impact on the overall BMI distribution^30^ and therefore, because our analyses cover the whole range of BMI levels, we think the overall pattern of association would not be markedly biased and nor would our estimate of a ‘healthy’ BMI. In addition, pregnant women might under-report smoking. Such misreporting may have resulted in residual confounding. Residual confounding could also be present if dietary patterns affected stillbirth and infant mortality. A Cochrane review found some evidence that balanced energy/protein supplementation vs no supplementation probably reduces stillbirth, but with no clear or equivalent effect of vitamin A or other nutrients, including multiple micronutrients vs iron supplementation with or without folic acid on stillbirth or perinatal death.^31^ Since micronutrient intake is unlikely to influence BMI, and effects on stillbirth/perinatal mortality are unclear, it is unlikely that these are major confounders of the associations we have observed. However, since the data are observational, we cannot assume higher or lower BMI causes these outcomes. It is not possible to randomize women to different BMI levels and whilst our recent Mendelian randomization analyses (discussed above) provides evidence of causal effects on outcomes such as hypertensive disorders of pregnancy and gestational diabetes that might mediate maternal BMI effects on stillbirth, infant and neonatal mortality, non-linear Mendelian randomization for these rare outcomes is currently unfeasible as no study or collaboration of studies would have sufficiently large numbers with genomic data and these outcomes. We used publicly available birth registration data which may contain recorded or other errors; however, the National Center for Health Statistics has systems for ongoing data quality improvements which should help to minimise such errors.^32^ Finally, our analysis is of women resident and pregnant in the US between 2014 and 2021 and may not generalise to other populations, particularly those at a different stage of the obesity epidemic. It has also been suggested that women from different ethnic backgrounds, in particular those of South Asian origin, have different body compositions that might influence their risk of stillbirth, neonatal and perinatal death. Whilst we previously found no evidence that using South Asian specific thresholds for obesity improved prediction of hypertensive disorders of pregnancy, gestational diabetes, macrosomia or preterm birth (all related to the mortality outcomes explored in this study) compared with conventional European derived thresholds,^33^ we cannot assume that the non-linear associations we have found here apply to low and middle income populations, where the majority of stillbirths and infant deaths occur, or to those from difference ethnic origins. If these results were replicated in different populations at different stages of the obesity epidemic and with different ethnic and socio-economic distributions that might increase our confidence in them being causal.

In conclusion, in this large contemporary high-income population we show that risk of stillbirth, infant mortality and neonatal mortality is increased at lower and higher maternal BMI, with pre-pregnancy BMI values around 21–22 kg/m^2^ likely to minimise risk for all three of these rare but devastating outcomes. Non-linear associations of maternal BMI are evident for birth weight, with higher BMI relating to large-for-gestational age and lower BMI linked to increased risk of small for gestational age, and for preterm birth.^34^ Taken together with results we present here, this emerging body of research highlights the need for public health advice to women of reproductive age to recognise that lower, as well as higher BMI is associated with adverse maternal and offspring outcomes.

Whilst we identified potential mediation of BMI associations with some of our outcomes by SGA, LGA and PTB, we do not feel that these findings are useful in terms of public health, where we would want to support women to have a healthy BMI prior to becoming pregnant to prevent stillbirth, infant and neonatal mortality. If there were effective means of preventing SGA, LGA and preterm birth then it could be argued that these interventions should be specifically targeted at women with a BMI outside the ‘healthy’ range as part of antenatal care. However, we are not aware of such interventions or their effects in women with lower and higher BMI and, as discussed above, conventional multivariable regression approaches to assessing mediation often violate the assumption of no residual mediator-outcome confounding, which if present will bias associations between exposure (here BMI) and outcome (stillbirth, infant and neonatal mortality) once the mediator is adjusted for.^22^ Future research should investigate whether the same patterns are observed in different populations, particularly low and middle-income countries, and in more diverse ethnic populations, aim to understand the factors and mechanisms which underlie these associations, and refine the range of BMI levels that optimise all aspects of maternal and offspring perinatal health.

## Contributors

DAL conceptualised the study, obtained the funding and supervised this work. DAL, RPC and HVT designed the study. RPC curated the data prior to analysis. HVT and RPC accessed and verified the underlying data and completed data analyses. HVT wrote the original draft; RC. RPC drafted the revisions. All three authors interpreted the data and contributed to reviewing and editing the manuscript and all act as guarantors for the research presented.

## Data sharing statement

NCHS Vital Statistics data are freely available (downloadable) at https://www.cdc.gov/nchs/data_access/vitalstatsonline.htm.

## Declaration of interests

DAL reports research grant funding from the UK Medical Research Council, the European Research Council, the UK National Institute of Health Research, the US National Institute of Health, the British Heart Foundation and Diabetes UK. The remaining authors declare they have nothing to disclose.
