# Supplementary material for: Non-linear associations of maternal pre-pregnancy body mass index with risk of stillbirth, infant, and neonatal mortality in over 28 million births in the USA: a retrospective cohort study
Source: eClinicalMedicine. 2023 Dec 2;66:102351. doi: 10.1016/j.eclinm.2023.102351 (PMC10730341; doi:10.1016/j.eclinm.2023.102351)
Supplement: Supplementary Material [file mmc1.pdf]

Supplementary material: Exploring non-linear associations of maternal pre-pregnant body mass index with risk of stillbirth, infant and neonatal mortality in over 28 million US births

A. Supplementary text

Fractional polynomial models

Fractional polynomial models are used to model non-linear relationships between an outcome and a continuous predictor [1].

For a binary outcome, they fit models of the form:

$logit(Y) = \beta_0 + \beta_1 X^{P_1}$ (one-degree model)

$logit(Y) = \beta_0 + \beta_1 X^{P_1} + \beta_2 X^{P_2}$ (two-degree model)

and so on,

where X is the continuous predictor, and P_1, P_2 (the powers) for each term are chosen from a small set of powers: {-2, 1, -0.5, 0, 0.5, 1, 2, 3}, where X^0 indicates ln(X), the natural logarithm of X.

In fractional polynomials, powers are allowed to repeat. Each time a power is repeated, it is multiplied by ln(X) – thus, if the best-fitting two-degree model had powers of e.g. 0.5 and 0.5, this would indicate terms in the square root of X and the natural logarithm of X multiplied by the square root of X.

The best-fitting model is chosen by comparing model deviances [1]. The deviance is measure of model fit, with smaller values representing a better fit [2].

Although models with more than two degrees are rarely needed [3], we investigated up to three degrees. To fit the fractional polynomials, BMI was first scaled [scaled BMI = (BMI-10)/5]. This is done because if the values of the variable are too large (or too small), this can generate extreme values with certain powers of this variable (e.g. cubic or squared reciprocal powers). The table below shows the mapping between BMI and scaled BMI.

Mapping between BMI and scaled BMI

BMI	15	20	25	30	35	40	45	50	55	60
Scaled BMI	1	2	3	4	5	6	7	8	9	10

Note: an alternative to fractional polynomials for modelling the relationship between a continuous covariate and the outcome of interest is restricted cubic splines. Cubic splines allow the modelling of some complex functions (e.g. curves with local minima or maxima) that are not possible with fractional polynomials. However, in the absence of such complexities, it has been shown that cubic splines and fractional polynomials often choose very similar models [4]. Because of this, and because fractional polynomials are generally easier to interpret, we decided to use these over cubic splines.

Results

For each outcome, the best fitting two-degree model provided a better fit than the best fitting one-degree model (difference in deviance comparing the two- to the one-degree model = 305.4 for infant mortality, 265.4 for neonatal mortality and 72.4 for stillbirth; $p<0.001$ for all three outcomes). For infant and neonatal mortality, the best fitting three-degree model provided a better fit than the best fitting two-degree model (difference in deviance comparing the three-degree to the two-degree model= 43.1 for infant mortality; 66.9 for neonatal mortality, $p<0.001$ in both cases). For stillbirth, there was a relatively small decrease in deviance (difference in deviance = 2.4, $p=0.3$) and the two-degree model was therefore used.

Supplementary Figures 1 and 2 compare the predicted risks of infant and neonatal mortality obtained using the best fitting two-degree and three-degree models. For both, the increased risk at both very low and very high BMIs were slightly lower with the three-degree model than the two-degree model. In addition, the curve started to flatten at BMIs above about 50 kg/m^2 and, for neonatal mortality, the estimated risk slightly decreased at the very highest BMIs, although the confidence interval was quite wide at this extreme. Finally, the confidence intervals were wider for high BMIs with the three-degree model compared to the two-degree model (Supplementary Figure 1 – infant mortality; Supplementary Figure 2 – neonatal mortality).

The final model for stillbirth had powers of -1, -0.5 (the reciprocal of scaled BMI, and the reciprocal of the square root of scaled BMI); for neonatal and infant mortality, the powers were 0.5, 0.5 and 0.5 (the square root of scaled BMI, the natural logarithm of scaled BMI multiplied by the square root of scaled BMI, and the natural logarithm of scaled BMI all squared multiplied by the square root of scaled BMI).

B. Supplementary tables and figures

Supplementary Table S1: Numbers and unadjusted rates (per 1,000 births) of stillbirth (24-week threshold, among analysis sample) by maternal pre-pregnant BMI categories

Maternal pre-pregnant BMI category	N	Number of stillbirths (rate /1,000 births)
Severe underweight: <16	65,066	211 (3.2)
Moderate underweight: 16 – 16.9	150,312	409 (2.7)
Mild underweight: 17 – 18.49	706,022	1,544 (2.2)
Normal weight: 18.5 – 24.9	12,087,906	26,697 (2.2)
Overweight: 25 – 29.9	7,481,922	20,535 (2.7)
Obesity class I: 30 – 34.9	4,230,384	13,988 (3.3)
Obesity class II: 35 – 39.9	2,091,080	7,766 (3.7)
Obesity class III: 40+	1,497,462	6,746 (4.5)

Supplementary Table S2: Numbers and unadjusted rates (per 1,000 live births) of infant and neonatal mortality (among analysis sample) by maternal pre-pregnant BMI categories

Maternal pre-pregnant BMI category	N	Number (rate/1,000 live births)	
		Infant mortality	Neonatal mortality
Severe underweight: <16	64,855	480 (7.4)	305 (4.7)
Moderate underweight: 16 – 16.9	149,902	910 (6.1)	541 (3.6)
Mild underweight: 17 – 18.49	704,465	3,650 (5.2)	2,268 (3.2)
Normal weight: 18.5 – 24.9	12,061,051	50,829 (4.2)	32,598 (2.7)
Overweight: 25 – 29.9	7,461,304	36,055 (4.8)	23,806 (3.2)
Obesity class I: 30 – 34.9	4,216,286	25,023 (5.9)	16,739 (4.0)
Obesity class II: 35 – 39.9	2,083,257	14,511 (7.0)	9,830 (4.7)
Obesity class III: 40+	1,490,687	12,162 (8.2)	8,159 (5.5)

Supplementary Table S3: Adjusted odds ratios (95% CI) for stillbirth, infant mortality and neonatal mortality by maternal pre-pregnant BMI categories

Maternal pre-pregnant BMI category	Stillbirth (24 weeks)	Infant mortality	Neonatal mortality
Severe underweight: <16	1.25 (1.09, 1.43)	1.39 (1.27,1.52)	1.48 (1.32, 1.67)
Moderate underweight: 16 – 16.9	1.08 (0.98, 1.19)	1.18 (1.11, 1.26)	1.17 (1.07, 1.29)
Mild underweight: 17 – 18.49	0.92 (0.87, 0.97)	1.06 (1.06, 1.13)	1.11 (1.06 (1.16)
Normal weight: 18.5 – 24.9	1.00	1.00	1.00
Overweight: 25 – 29.9	1.17 (1.15, 1.20)	1.08 (1.06, 1.09)	1.12 (1.10, 1.14)
Obesity class I: 30 – 34.9	1.35 (1.32, 1.37)	1.24 (1.22, 1.26)	1.31 (1.29, 1.34)
Obesity class II: 35 – 39.9	1.47 (1.44, 1.51)	1.39 (1.36, 1.42)	1.49 (1.46, 1.53)
Obesity class III: 40+	1.73 (1.68, 1.78)	1.54 (1.51, 1.57)	1.63 (1.59, 1.67)

Supplementary Table S4: Adjusted odds ratios (95% CI) for stillbirth using the 20-week and 28-week thresholds

Maternal pre-pregnant BMI category	Stillbirth (20 weeks)	Stillbirth (28 weeks)
Severe underweight: <16	1.25 (1.11, 1.39)	1.17 (1.00, 1.38)
Moderate underweight: 16 – 16.9	1.13 (1.04, 1.23)	1.04 (0.93, 1.17)
Mild underweight: 17 – 18.49	0.96 (0.92, 1.00)	0.89 (0.84, 0.95)
Normal weight: 18.5 – 24.9	1.00	1.00
Overweight: 25 – 29.9	1.16 (1.15, 1.18)	1.18 (1.16, 1.21)
Obesity class I: 30 – 34.9	1.36 (1.34, 1.39)	1.35 (1.32, 1.38)
Obesity class II: 35 – 39.9	1.53 (1.50, 1.56)	1.50 (1.46, 1.55)
Obesity class III: 40+	1.75 (1.71, 1.80)	1.73 (1.68, 1.79)

Supplementary Figure S1: Predicted risk of infant mortality by maternal pre-pregnant BMI comparing the best fitting two-degree and three-degree models (shaded area indicates 95% confidence interval)

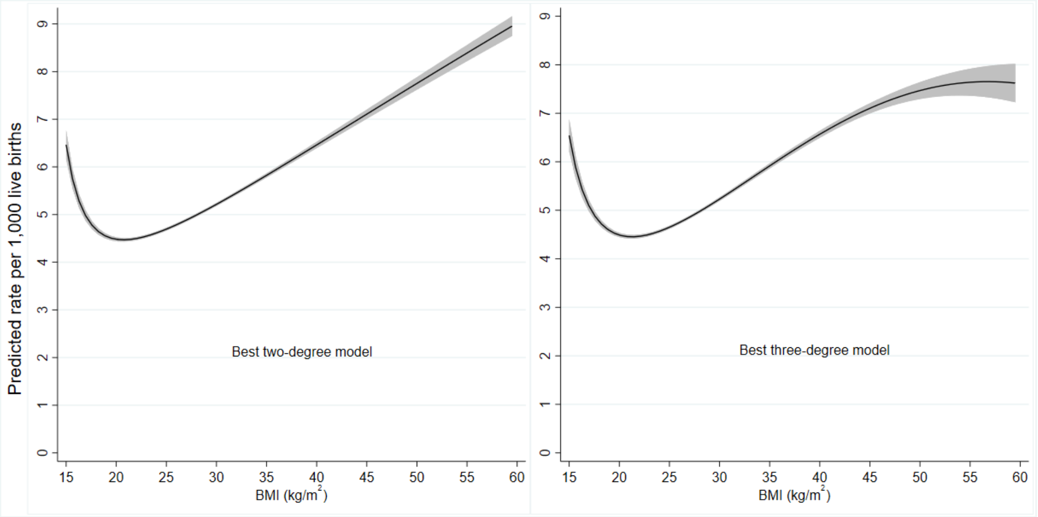

Supplementary Figure S2: Predicted risk of neonatal mortality by maternal pre-pregnant BMI comparing the best fitting two-degree and three-degree models (shaded area indicates 95% confidence interval)

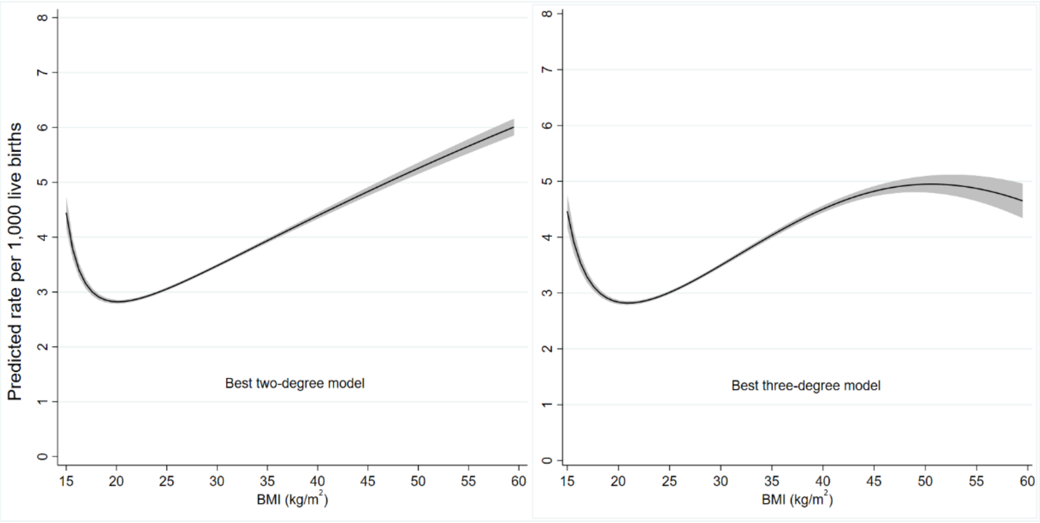

Supplementary Figure S3: Predicted risk of stillbirth by maternal pre-pregnant BMI for the different stillbirth definitions (shaded area indicates 95% confidence interval)

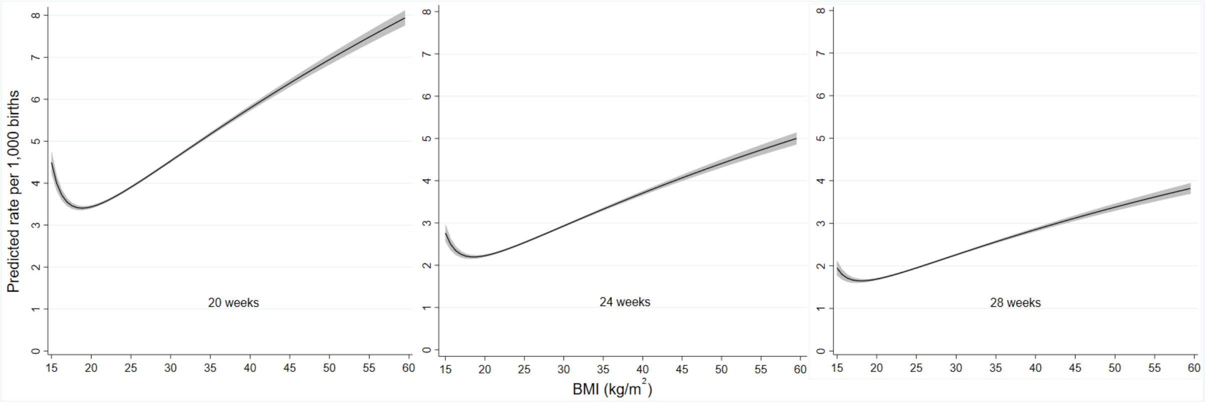

Supplementary Figure S4: Relationship between maternal pre-pregnant BMI and stillbirth after including mediators in the model (shaded area indicates 95% confidence interval)

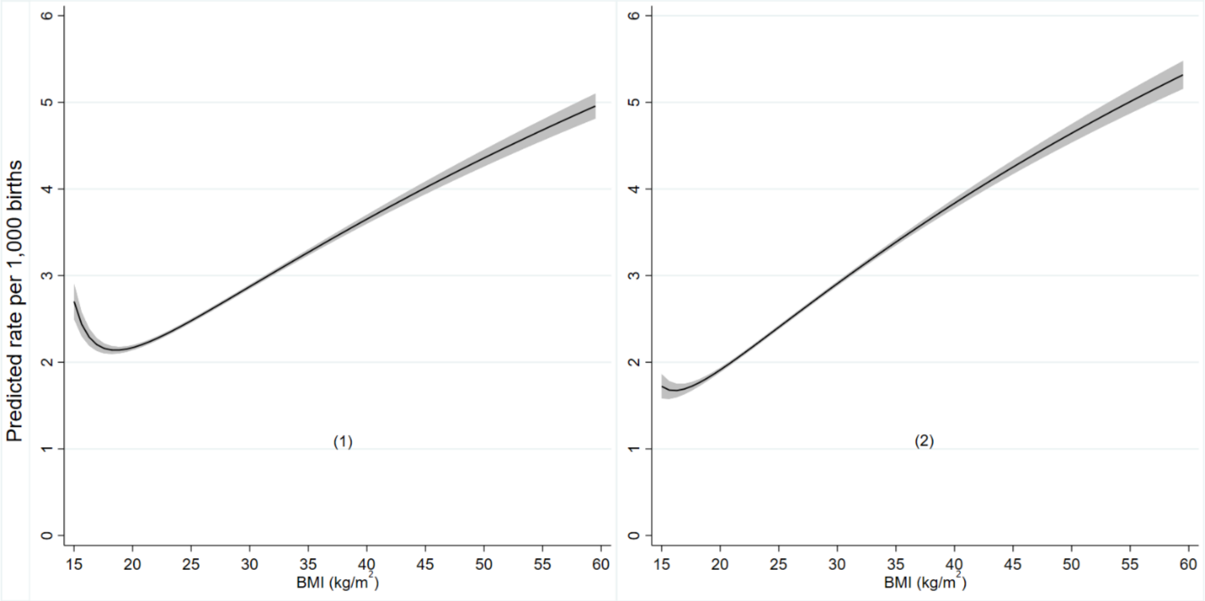

(1) Model including confounders plus gestational diabetes, gestational hypertension and pre-eclampsia
(2) Model additionally including SGA and LGA

Supplementary Figure S5: Relationship between maternal pre-pregnant BMI and infant and neonatal mortality after including mediators in the model (shaded area indicates 95% confidence interval)

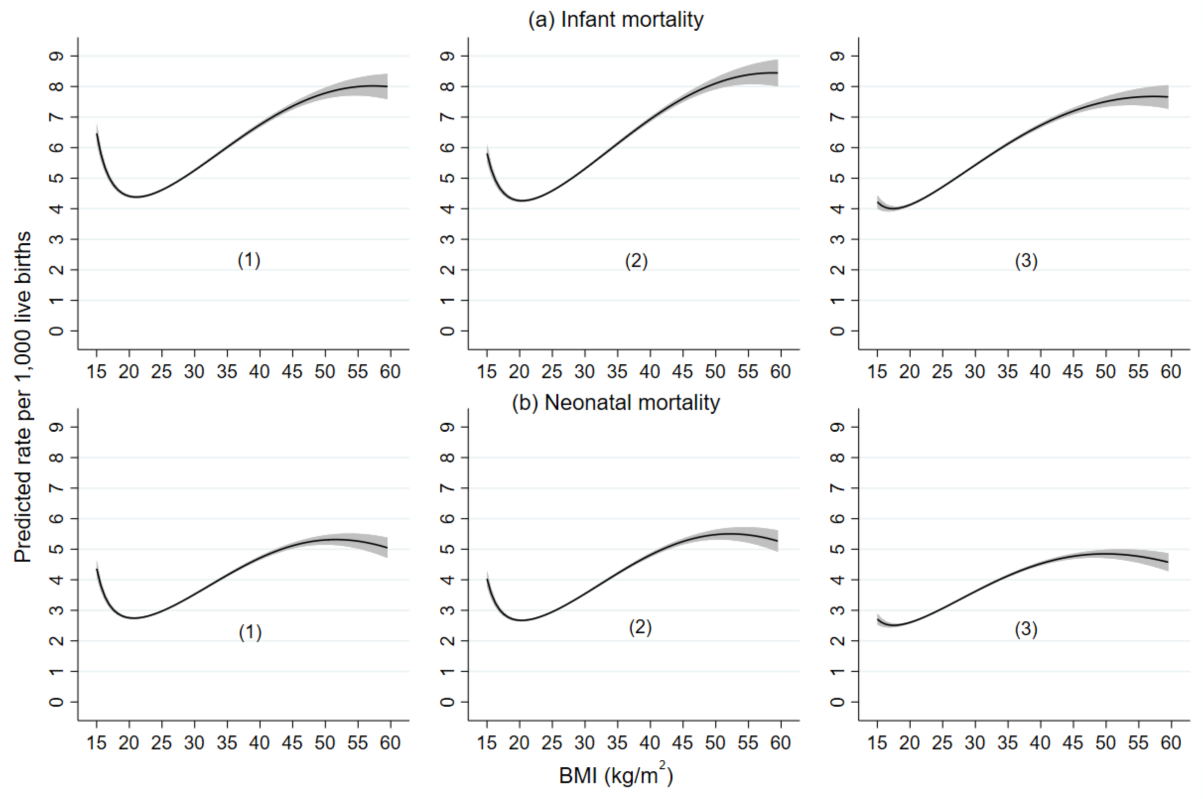

(3) Model including confounders plus gestational diabetes, gestational hypertension and pre-eclampsia
(4) Model additionally including SGA and LGA
(5) Model additionally including preterm birth

Supplementary Table S5: Adjusted odds ratios (95% CI) for stillbirth, infant mortality and neonatal mortality by maternal pre-pregnant BMI categories, with no adjustment for potential mediating factors, additional adjustment for gestational hypertension, gestational diabetes and pre-eclampsia, additional adjustment for SGA and LGA and, for infant and neonatal mortality only, additional adjustment for preterm birth (<37 completed weeks gestation)

Maternal pre-pregnant BMI category	Adjusted for confounders only[1]	Adjusted for confounders + gestational diabetes, gestational hypertension & pre-eclampsia	Additionally adjusted for SGA and LGA	Additionally adjusted for preterm birth
Stillbirth (24 weeks)				
Severe underweight	1.25 (1.09, 1.43)	1.24 (1.08, 1.43)	0.94 (0.82, 1.08)	Not applicable
Moderate underwgt	1.08 (0.98, 1.19)	1.07 (0.97, 1.19)	0.86 (0.78, 0.96)	
Mild underweight	0.92 (0.87, 0.97)	0.92 (0.87, 0.97)	0.78 (0.74, 0.83)	
Normal weight	1.00	1.00	1.00	
Overweight	1.17 (1.15, 1.20)	1.18 (1.16, 1.20)	1.28 (1.25, 1.30)	
Obesity class I	1.35 (1.32, 1.37)	1.35 (1.32, 1.38)	1.50 (1.47, 1.53)	
Obesity class II	1.47 (1.44, 1.51)	1.49 (1.45, 1.53)	1.67 (1.63, 1.72)	
Obesity class III	1.73 (1.68, 1.78)	1.75 (1.70, 1.80)	2.02 (1.96, 2.08)	
Infant mortality				
Severe underweight	1.39 (1.27, 1.52)	1.38 (1.26, 1.52)	1.29 (1.17, 1.41)	1.00 (0.91, 1.10)
Moderate underwgt	1.18 (1.11, 1.26)	1.17 (1.10, 1.26)	1.11 (1.04, 1.19)	0.89 (0.84, 0.96)
Mild underweight	1.06 (1.06, 1.13)	1.09 (1.05, 1.13)	1.05 (1.01, 1.08)	0.92 (0.88, 0.95)
Normal weight	1.00	1.00	1.00	1.00
Overweight	1.08 (1.06, 1.09)	1.09 (1.07, 1.10)	1.12 (1.10, 1.13)	1.15 (1.13, 1.17)
Obesity class I	1.24 (1.22, 1.26)	1.27 (1.25, 1.29)	1.31 (1.29, 1.33)	1.32 (1.30, 1.35)
Obesity class II	1.39 (1.36, 1.42)	1.44 (1.41, 1.47)	1.51 (1.48, 1.54)	1.50 (1.47, 1.53)
Obesity class III	1.54 (1.51, 1.57)	1.62 (1.58, 1.65)	1.71 (1.67, 1.74)	1.65 (1.61, 1.68)
Neonatal mortality				
Severe underweight	1.48 (1.32, 1.67)	1.48 (1.32, 1.67)	1.40 (1.25, 1.58)	1.02 (0.90, 1.15)
Moderate underwgt	1.17 (1.07, 1.29)	1.17 (1.07, 1.29)	1.11 (1.02, 1.21)	0.84 (0.77, 0.92)
Mild underweight	1.11 (1.06 (1.16)	1.11 (1.06 (1.16)	1.07 (1.02 (1.12)	0.91 (0.87, 0.95)
Normal weight	1.00	1.00	1.00	1.00
Overweight	1.12 (1.10, 1.14)	1.12 (1.10, 1.14)	1.16 (1.14, 1.18)	1.19 (1.17, 1.21)
Obesity class I	1.31 (1.29, 1.34)	1.31 (1.29, 1.34)	1.41 (1.38, 1.44)	1.39 (1.36, 1.42)
Obesity class II	1.49 (1.46, 1.53)	1.49 (1.46, 1.53)	1.65 (1.61, 1.69)	1.58 (1.54, 1.62)
Obesity class III	1.63 (1.59, 1.67)	1.63 (1.59, 1.67)	1.85 (1.80, 1.90)	1.70 (1.66, 1.75)

1. Note that the first set of results (adjusted for confounders only) are the same as those shown in Supplementary Table S3; they are repeated in this table to facilitate comparison with the other model results

C. Stata code

Below is the code for the stillbirth analysis. The code for infant or neonatal mortality was the same barring the name of the outcome variable and the powers of the fractional polynomials (and clearly without the additional analyses using the different definitions of stillbirth).

```
** Stillbirth analysis
use births2014to2021, clear
log using stillbirths.log
egen missing = rowmiss(bmi magegp medgp methnic smokepreg bintgp12m parity multiplebirth death28)
gen complete=missing==0

*Table of characteristics - all + complete cases
tab1 death24 bmigp magegp medgp methnic smokepreg parity bintgp12m multiplebirth
tab1 death24 bmigp magegp medgp methnic smokepreg parity bintgp12m multiplebirth if complete==1

*Risk of stillbirth by BMI categories (extended underweight groups)
tab death24 bmigp8 if complete==1, col

*Adjusted and unadjusted ORs for BMI categories (extended underweight groups)
logistic death24 ib1.bmigp8 if complete==1, vce (cluster pregid)
logistic death24 ib1.bmigp8 ib2.magegp i.medgp i.methnic i.smokepreg i.bintgp12m i.parity i.multiplebirth, vce
(cluster pregid)

*Repeat adjusted logistic regression for 20-week and 28-week thresholds
logistic death20 ib1.bmigp8 ib2.magegp i.medgp i.methnic i.smokepreg i.bintgp12m i.parity i.multiplebirth, vce
(cluster pregid)
logistic death28 ib1.bmigp8 ib2.magegp i.medgp i.methnic i.smokepreg i.bintgp12m i.parity i.multiplebirth, vce
(cluster pregid)

**Generate scaled bmi for fractional polynomials
gen bmi5=(bmi-10)/5
*Comparison of models:
fp <bmi5>, dim(3) replace: logistic death24 <bmi5> i.parity ib2.magegp i.smokepreg i.methnic i.medgp
i.bintgp12m multiplebirth
*Fit final model using robust standard errors
fp <bmi5>, fp(-1 -1 3) replace: logit death24 <bmi5> i.parity ib2.magegp i.smokepreg i.methnic i.medgp
i.bintgp12m multiplebirth, vce(cluster pregid)
*Graph of predicted risk
marginscontplot bmi5 (bmi5_1 bmi5_2 bmi5_3), at1(1(0.129)10) ci
*Best fitting two-degree model for comparison
fp <bmi5>, fp(-1 -0.5) replace: logit death24 <bmi5> i.parity ib2.magegp i.smokepreg i.methnic i.medgp
i.bintgp12m multiplebirth, vce(cluster pregid)
marginscontplot bmi5 (bmi5_1 bmi5_2), at1(1(0.129)10) ci

*Plots for 20-week and 28-week thresholds
fp <bmi5>, fp(-1 -1 3) replace: logit death20 <bmi5> i.parity ib2.magegp i.smokepreg i.methnic i.medgp
i.bintgp12m multiplebirth, vce(cluster pregid)
*Graph of predicted risk
marginscontplot bmi5 (bmi5_1 bmi5_2 bmi5_3), at1(1(0.129)10) ci
fp <bmi5>, fp(-1 -1 3) replace: logit death28 <bmi5> i.parity ib2.magegp i.smokepreg i.methnic i.medgp
i.bintgp12m multiplebirth, vce(cluster pregid)
*Graph of predicted risk
marginscontplot bmi5 (bmi5_1 bmi5_2 bmi5_3), at1(1(0.129)10) ci
```

```
*Obtain coefficients and variance-covariance matrix for bootstrapping
fp <bmi5>, fp(-1 -1 3) replace: logit death24 <bmi5> i.parity ib2.magegp i.smokepreg i.methnic i.medgp i.bintgp12m multiplebirth, vce(cluster pregid)
matrix coeff = get(_b)
matrix V = get(VCE)
*Bootstrapping to obtain 95% CI for BMI where risk is minimum
drawnorm b1 b2 b3 b4 b5 b6 b7 b8 b9 b10 b11 b12 b13 b14 b15 b16 b17 b18 b19 b20 b21 b22 b23 b24 b25 b26 b27 b28 b29 b30 cons, n(100000) means(coeff) cov(V) clear
save stillbirth_minsim, replace
_pctile b1, p(2.5, 50, 97.5)
return list
_pctile b2, p(2.5, 50, 97.5)
return list
_pctile b3, p(2.5, 50, 97.5)
return list

*Including potential mediators
fp <bmi5>, fp(-1 -1 3) replace: logit death24 <bmi5> i.parity ib2.magegp i.smokepreg i.methnic i.medgp i.bintgp12m multiplebirth gdiabetes ghyper preeclamp, vce(cluster pregid)
fp <bmi5>, fp(-1 -1 3) replace: logit death24 <bmi5> i.parity ib2.magegp i.smokepreg i.methnic i.medgp i.bintgp12m multiplebirth gdiabetes ghyper preeclamp sga lga, vce(cluster pregid)
logistic death24 ib1.bmigp8 ib2.magegp i.medgp i.methnic i.smokepreg i.bintgp12m i.parity i.multiplebirth gdiabetes ghyper preeclamp, vce (cluster pregid)
logistic death24 ib1.bmigp8 ib2.magegp i.medgp i.methnic i.smokepreg i.bintgp12m i.parity i.multiplebirth gdiabetes ghyper preeclamp sga lga, vce (cluster pregid)
```

References

1. Royston P, Ambler G, Sauerbrei W. The use of fractional polynomials to model continuous risk variables in epidemiology. Int J Epidemiol 1999;28(5):964-974. https://doi.org/10.1093/ije/28.5.964
2. Kutner MH, Nachtsheim CJ, Neter J, Li W. Applied linear statistical models. Fifth Edition. New York (USA): McGraw-Hill; 2005.
3. Royston P and Altman DG. Regression using fractional polynomials of continuous covariates: parsimonious parametric modelling. J Royal Stat Soc Series C (Applied Statistics) 1994; 43(3):*429*–67. https://doi.org/10.2307/2986270
4. Binder H, Sauerbrei W, Royston P. Comparison between splines and fractional polynomials for multivariable model building with continuous covariates: a simulation study with continuous response. Statist Med 2013; 32:2262-77. https://doi.org/10.1002/sim.5639
